# A Novel Strategy to Screen Bacillus Calmette-Guérin Protein Antigen Recognized by γδ TCR

**DOI:** 10.1371/journal.pone.0018809

**Published:** 2011-04-22

**Authors:** XueYan Xi, XiaoYan Zhang, Bei Wang, Ji Wang, He Huang, LianXian Cui, XiQin Han, Liang Li, Wei He, ZhenDong Zhao

**Affiliations:** 1 State Key Laboratory for Molecular Virology and Genetic Engineering, Institute of Pathogen Biology, Chinese Academy of Medical Sciences and Peking Union Medical College, Beijing, China; 2 Department of Immunology, Institute of Basic Medical Sciences, Chinese Academy of Medical Sciences and Peking Union Medical College, Beijing, China; 3 Beijing Tuberculosis and Thoracic Tumor Research Institute, Beijing, China; Fundação Oswaldo Cruz, Brazil

## Abstract

**Background:**

Phosphoantigen was originally identified as the main γδ TCR-recognized antigen that could activate γδ T cells to promote immune protection against mycobacterial infection. However, new evidence shows that the γδ T cells activated by phosphoantigen can only provide partial immune protection against mycobacterial infection. In contrast, whole lysates of *Mycobacterium* could activate immune protection more potently, implying that other γδ TCR-recognized antigens that elicit protective immune responses. To date, only a few distinct mycobacterial antigens recognized by the γδ TCR have been characterized.

**Methodology/Principal Findings:**

In the present study, we established a new approach to screen epitopes or protein antigens recognized by the γδ TCR using Bacillus Calmette-Guérin- (BCG-) specific γ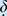 TCR transfected cells as probes to pan a 12-mer random-peptide phage-displayed library. Through binding assays and functional analysis, we identified a peptide (BP3) that not only binds to the BCG-specific γδ TCR but also effectively activates γδ T cells isolated from human subjects inoculated with BCG. Importantly, the γδ T cells activated by peptide BP3 had a cytotoxic effect on THP-1 cells infected with BCG. Moreover, the oxidative stress response regulatory protein (OXYS), a BCG protein that matches perfectly with peptide BP3 according to bioinformatics analysis, was confirmed as a ligand for the γδ TCR and was found to activate γδ T cells from human subjects inoculated with BCG.

**Conclusions/Significance:**

In conclusion, our study provides a novel strategy to identify epitopes or protein antigens for the γδ TCR, and provides a potential means to screen mycobacterial vaccines or candidates for adjuvant.

## Introduction

Approximately 2 billion people worldwide are infected with *M. tuberculosis* and around 3 million deaths occur annually due to this disease. Several immune protective mechanisms are involved in controlling the infection of *M. tuberculosis* including cytokine release and effector functions of immune cells. γ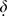 T cells, a subset of immune cells, play important roles in host immunity as a bridge between innate and adaptive immunity. In recent years, increasing amounts of research have concentrated on the immune protective function of γδ T cells in *M. tuberculosis* infection [1]–[4]. However, the mechanisms by which γδ T cells recognize tuberculosis antigens remain unclear and little is known about the distinct tuberculosis protein antigens that can effectively activate γ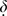 T cells.

γ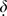 T cells recognize a variety of antigens. The best-known antigens for γ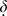 T cells are phosphoantigens, such as pyrophosphoantigen, aminobisphonate, and alkylamine [5]–[6]. With the progress of research in γδ T cells, additional protein antigens recognized by γδ T cells are being identified continually. MHC class I chain-related gene A (MICA) and B (MICB) were recognized by Vδ1 γ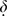 T cells [7]. UL-16 binding protein 4 (ULBP4) was shown to be a novel ligand for γ9/δ2 T cells [8]. The results of Chen *et al.* [9] suggested that human mutS homolog 2 (hMSH2) might be a new ligand for the γδ TCR. Moreover, other protein antigens, such as ectopically expressed mitochondrial ATPase [10], were identified as ligands for the γδ TCR.

γδ T cells recognize *M. tuberculosis* antigen and promote immune-protection against mycobacterial infection. Originally, phosphoantigen was regarded as the main γδ TCR-recognized antigen that activated γδ T cells. However, phosphoantigen- activated γδ T cells display a restricted TCR diversity and only a subset of phosphoantigen-responsive γδ T cells mediate protective immunity against tuberculosis [11]. In contrast, whole lysates of *Mycobacterium* activate immune protection more potently, implying that other γδ TCR-recognized antigens in *Mycobacterium* also elicit protective immune responses [4]. Boom *et al.* showed that protein antigens with a molecular mass of 10–14 kDa in the supernatant from heat-treated *Mycobacterium* functioned as bioactivators that stimulated γδ T cells [12]. The protein antigens of *M. tuberculosis* effectively activated γδ T cells, which in turn induced innate and adaptive immunity to *M. tuberculosis*. At the same time, γ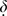 T cells participated in the anti-tuberculosis immune response elicited by other immune cells [13]–[16]. The interaction net composed of many types of immune cells plays an important role in the control of *M tuberculosis*. However, up to now, ESAT-6 [17] is the only tuberculosis protein antigens identified to be recognized by γδ TCR.

Recently, a novel strategy that used intact cells as probes to pan target peptides in a phage-display library was developed [18]–[19]. Using the hepatocellular carcinoma cell line HepG2 as a probe, Zhang *et al.* obtained a target peptide HCBP1 that may be a potential candidate for targeted drug therapy for liver cancer [20]. To identify mycobacterial antigen, Alderson *et al.* developed a technique using *Mycobacterium*-specific CD4^+^ T cells to screen a mycobacterial genomic library. The direct identification of antigens using T cells would undoubtedly be critical to detect unknown antigens in *Mycobacterium* [21]. Their research inspired us to develop a novel strategy to screen a phage-display library using γδ T cells cultured *in vitro*. To obtain γδ T cells purely by *in vivo* culture was infeasible due to the difficulty in culturing these cells and the limited quantity of γδ T cells. We addressed these problems by transfecting cells with specific γδ TCR. After the full-length γ chain and δ chain were co-transfected to J.RT3-T3.5 cells, a T-lymphoma cell line deficient in both TCR α/γ and β/δ chains (ATCC), the transfected cells with homogeneous γδ TCR were used as γδ T cells to analyze the recognition mechanism of γδ TCR to antigens [8], [22], [23], [24] and to identify the antigens recognized by γδ T cells [8]. In addition, while the antigen binding site of the γδ TCR is primarily formed from 3 CDRs contributed by each Vγ or Vδ domain, the sequence diversity in antigen receptors is highly concentrated in 1 or 2 CDR3s [9]. Based on known CDR3 sequences of γδ T cells, we generated cell lines expressing specific γδ TCR, which may be used as a probe to pan antigen peptides recognized by γδ TCR.

In this study, the BCG-specific γδ TCR-expressing cell lines (tester) and non-specific γδ TCR (driver) were used to carry out subtractive screening *in vitro* with a phage display 12-mer peptide library. After 4 rounds of panning, there was an obvious enrichment for the phages specifically binding to the γδ TCR. A group of peptides capable of specifically binding to the γδ TCR was obtained. After functional tests and bioinformatics analyses, we identified a target peptide (BP3) that could be recognized by the γδ TCR and activate γδ T cells from human subjects inoculated with BCG. Importantly, we demonstrate that oxidative stress response regulatory protein (OXYS) that contains the identified peptide could be recognized by γδ TCR and activate γδ T cells from human subjects inoculated with BCG.

## Results

### Construction of the transfected cells expressing BCG specific γ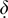 TCR (BCG γδ T cells) and BCG non-specific γδ TCR (DBS4.3 γδ T cells)

**Figure 1:**
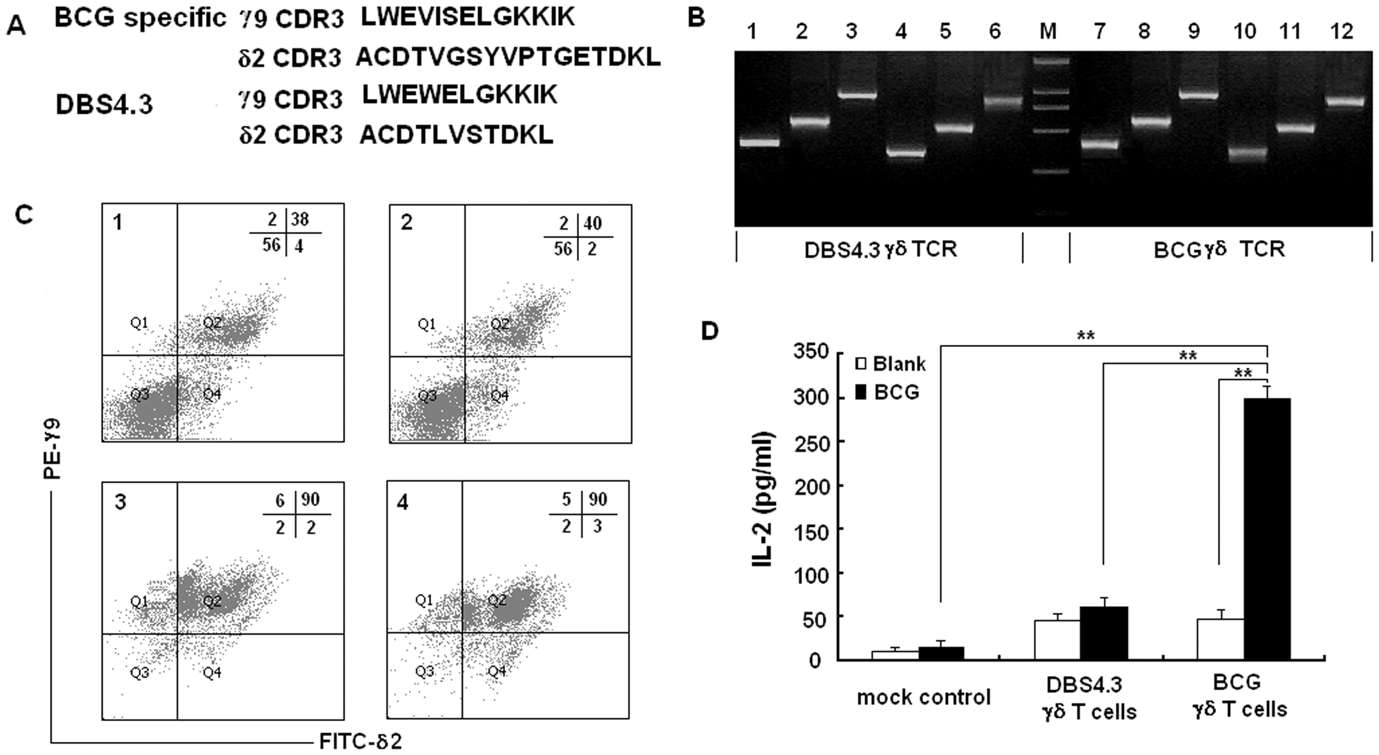
Construction of the transfected cells expressing BCG specific γδ TCR and DBS 4.3 γδ TCR. (A) The prominent γ9 and δ2 CDR3 sequence of BCG specific γδ TCR and DBS4.3 γδ TCR in human. (B) Construction of full-length γ9 and δ2 chain with BCG specific CDR3 sequence and DBS4.3 γδ TCR by overlapping PCR. Lane 1 and lane 7. The PCR product amplified by using γ9 upstream primer and overlapping reverse primer; Lane 2 and lane 8. The PCR product amplified by using overlapping forward primer and γ9 downstream primer; Lane 3 and lane 9. The full-length γ9 chain containing prominent BCG specific CDR3 sequence or DBS4.3 CDR3 sequence amplified by using above PCR products as the template and γ9 upstream and downstream primer as the primers; Lane 4 and lane 10. The PCR product amplified by using δ2 upstream primer and overlapping reverse primer; Lane 5 and lane 11. The PCR product amplified by using overlapping forward primer and δ2 downstream primer; Lane 6 and lane 12. The full-length of δ2 chain with prominent BCG specific CDR3 sequence or DBS4.3 CDR3 sequence amplified by using above PCR products as the template and δ2 upstream and downstream primer as the primers. (C) Assessment of γδ TCR expressed on J.RT3-T3.5 transfected cells via FACS analysis. Cells were stained with PE-anti γ9 monoclonal antibody and FITC-anti δ2 TCR monoclonal antibody. Panel 1 and panel 3 were the transfected cells expressing BCG specific γδ TCR before and after flow sorting, respectively. Panel 2 and panel 4 were the transfected cells expressing DBS4.3 γ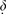 TCR before and after flow sorting, respectively. (D) IL-2 secretion of the BCG specific γδ TCR and DBS 4.3 γδ TCR stimulated with whole protein extracts of BCG were measured by ELISA. The double asterisks denote significant difference (p<0.01). Data are shown as the mean of pg/ml±S. D. of three independent experiments.

As reported by Spencer [11], the dominant BCG specific γ9 CDR3 sequence in human is LWEVISELGKKIK and δ2 CDR3 is ACDTVGSYVPTGETDKL. Accordingly, we designed the overlapping forward and reverse primer (supporting information table S1) containing the nucleic acid sequences encoding the above peptides to substitute the original CDR3 region by using overlapping PCR. The PCR products of the full-length γ9 and δ2 chains are shown in Figure 1B. These PCR fragments were then cloned into pREP7 and pREP9 expression vectors and co-transfected into J.RT3-T3.5 cells by electroporation. After selection and FACS analysis, ∼40% of the transfected cells were shown to express the γ9/δ2 TCR (Figure 1C). We chose a known human γδ T cell clone, DBS4.3 γδ T cells to be the BCG non-specific γδ TCR control. The γ9 and δ2 CDR3 sequences of DBS4.3 are LWEWELGKKIK and ACDTLVSTDKL [26], respectively (Figure 1A). The cells expressing DBS4.3 γδ TCR were constructed using a similar procedure. Forty percent of the cells expressed the γ9/δ2 TCR. The transfected cells were enriched by flow sorting (Figure 1C). To check the BCG specificity of the transfected γδ TCR, the cells expressing BCG-specific and non-specific γδ TCR were stimulated with protein extracts of BCG and the level of secreted IL-2 was measured by ELISA. As shown in Figure 1D, the protein extract of BCG stimulated the BCG-specific γδ T cells and induced much more IL-2 than the DBS4.3 γδ T cells, suggesting that J.RT3-T3.5 transfected cells expressing BCG-specific γδ TCR were successfully generated.

### Biopanning of BCG γ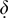 TCR recognized peptides from phage library using the transfected cells

**Figure 2:**
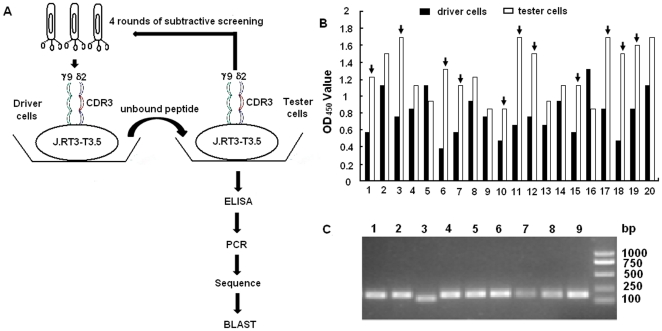
Biopanning of BCG specific γδ TCR recognized peptides from phage library using the transfected cells. (A) The flow chart about screening and analysis of the peptides recognized by BCG specific γδ TCR. (B) Selection of BCG specific γδ TCR recognized positive phage clones by phage-ELISA. Representative data are shown in this figure. Dark arrow pointed bars represent the ratio of specific binding to nonspecific binding was more than 2, and corresponding phage clones were taken as positive clones. (C) Confirmation of BCG specific γδ TCR recognized positive clones by RT-PCR. The BCG specific γδ TCR recognized peptides were rescued by RT-PCR using phage specific primer provided in kit. 9 individual representative clones are shown in this figure. The RT-PCR products of positive clones were sequenced using specific sequencing primer.

To identify the epitopes recognized by BCG-specific γ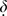 TCR, we performed a γδ TCR cell-mediated biopanning of a 12-mer random peptide phage-display library. The experimental procedure is shown in Figure 2A. Phages specifically binding to BCG-specific γδ TCR were identified through 4 rounds of *in vitro* panning. After 4 rounds of *in vitro* selection and enrichment, the number of eluted phages increased 4000 fold (from 5.8×10^4^ pfu to 2.4×10^8^ pfu) compared to that of the phages in the first round (Table 1). The output/input ratio of phages after each round of panning was used to determine the phage recovery efficiency and an obvious enrichment of phages specifically binding to BCG γδ T cells was found (Table 1). By analyzing the results of phage-ELISA (Figure 2B), 30 phage clones specifically binding to the BCG-specific γδ T cells (the ratio of specific binding to nonspecific binding was more than 2) were amplified by RT-PCR (Figure 2C). The RT-PCR products were sequenced using the specific sequencing primer provided from the kit and the results are shown in Table 2. Three prominent peptides were selected as candidates for further functional study based on their high frequency of appearance among the 30 sequences. These three peptides were designated as BP1, BP2, and BP3 respectively and then chemically synthesized for further use.

**Table 1:** Output Assay for Each Round of Plaque Selection.

Round of bio-panning	Phages inputted(PFU*)	Phages eluted(PFU)	Yield**
1st	1.5×10^11^	5.8×10^4^	3.8×10^−7^
2nd	2.5×10^11^	2.8×10^6^	1.1×10^−5^
3rd	3.0×10^11^	2.2×10^8^	7.3×10^−4^
4th	2.0×10^11^	2.4×10^8^	1.2×10^−3^

*PFU:Plaque forming unit.

**Yield = phages eluted/phages inputted.

**Table 2:** The Amino Acid Sequences of 12 mer Peptides Screened from the Phage Library.

Phage clones	Name	Sequence	Frequency
12/14/19/20/21/24/27/28/30	BP1	VSRHQSWHPHDL	9/30
4/10/17/26/29	BP2	KPLSKELTLPLQ	5/30
2/8/13/15/16	BP3	HPETQDSDDVDR	5/30
23/25	BP4	ALQLDPTPGPLR	2/30
18/22	BP5	VQPPPKSILGVA	2/30
6/7	BP6	HKLPVTMVLDRA	2/30
11	BP7	SHVDDIQRIPSS	1/30
5	BP8	TSAHGVPIHDYG	1/30
9	BP9	YTPQLLSETSYF	1/30
1	BP10	NFCSYQTYGHIT	1/30
3	BP11	GHTYRPTPSSLL	1/30

### Confirmation of peptide binding to the BCG specific γδ TCR transfected cells

**Figure 3:**
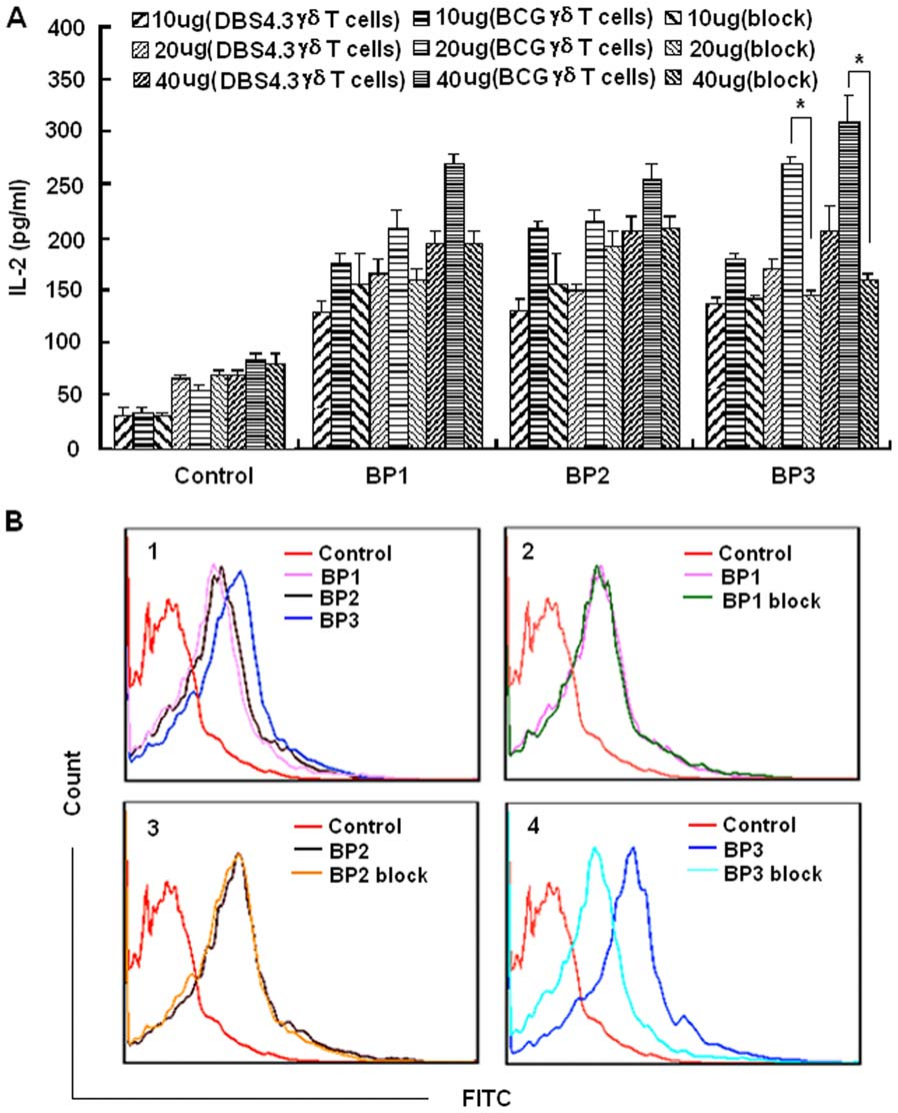
Confirmation of the peptide specifically binding to the BCG γδ TCR transfected cells. (A) Peptide binding and blocking assay by detecting the production of IL-2. The DBS4.3 γδ T cells, BCG γδ T cells and BCG γδ T cells blocked with functional γδ TCR blocking monoclonal antibody were stimulated by correspondent epitopic peptides at different concentration, secreted IL-2 was detected by ELISA. Data are shown as the mean of pg/ml±S.D. of three independent experiments. The single asterisk denote significant difference (p<0.05). (B) Peptide binding and blocking assay by FACS. The binding percentage of BCG γδ T cells or pre-blocked cells by functional γδ TCR blocking monoclonal antibody with biotinylated peptides was evaluated by FACS. The biotinylated peptides were co-cultured with BCG γδ T cells or pre-blocked cells by functional γδ TCR blocking monoclonal antibody at 4°C for 1 hour. FITC-coupled streptavidin was added. FACS assay were performed to detect the binding percentage of those γδ T cells with peptides. Panel 1 was the binding percentage of control peptide and three peptides with BCG γδ T cells. Panel 2, panel 3 and panel 4 were the binding percentage of BP1, BP2 and BP3 with BCG γδ T cells or pre-blocked BCG γδ T cells, respectively.

Binding assays and blocking assays were performed to determine whether the synthesized peptides specifically bind to BCG-specific γδ TCR, using ELISA and FACS. We first examined IL-2 production after the transfected cells were stimulated by the 3 putative peptides. All 3 peptides could stimulate BCG-specific γδ T cells to secrete IL-2 in a dose-dependent manner. Importantly, the IL-2 production of the BCG-specific γδ T cells stimulated by BP3 could be significantly blocked by functional γδ TCR blocking monoclonal antibody at some dosages, suggesting that the binding between BP3 and the BCG-specific γδ TCR transfected cells was specific (Figure 3A). The binding of transfected cells with peptides was further confirmed by FACS analysis. Although BP1, BP2, and BP3 all bound to BCG-specific γδ T cells, only the binding of BP3 could be blocked by functional γδ TCR blocking monoclonal antibody (Figure 3B). These results suggest that BP3, as a candidate epitope peptide, specifically bound to BCG-specific γδ T cells.

### Binding and activation of γδ T cells from human subjects inoculated with BCG by epitope peptides *in vitro*

**Figure 4:**
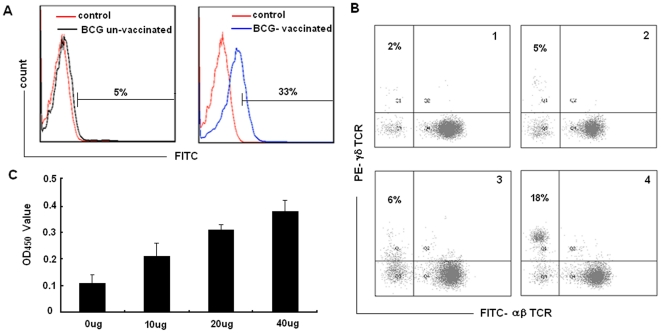
Binding and activating γδ T cells from BCG vaccinated and BCG un-vaccinated persons by epitope peptides *in vitro*. (A) BP3 could bind γδ T cells from human subjects inoculated with BCG *in vitro*. Fresh PBMC from BCG vaccinated and BCG un-vaccinated persons in 24-well culture plates with RPMI 1640 medium were immobilized with anti-pan γδ TCR monoclonal antibody for two weeks to obtain enough amounts of γδ T cells. The cells incubated with biotin-conjugated peptide or control peptide and FITC-conjugated streptavidin, and then subjected to FACS analysis. Results are representative of three independent experiments. (B) The immobilized BP3 was able to induce expansion of γδ T cells from human subjects inoculated by BCG. PBMC from BCG vaccinated and BCG un-vaccinated persons were cultured in 24-well plates with immobilized BP3 and control peptide in RPMI 1640 medium with 10% FCS and IL-2 (200units/ml). The percentage of γδ T cells was measured 2 weeks later by FACS. Results are representative of three independent experiments. Panel 1 and panel 2 was the proliferation percentage of γδ T cells from human subjects un-inoculated with BCG and inoculated with BCG induced by control peptide, respectively. Panel 3 and panel 4 was the proliferation percentage of γδ T cells from human subjects un-inoculated with BCG and inoculated with BCG induced by BP3, respectively. (C) Detection of γδ T cell proliferation by CCK-8 assay. After the γδ T cells expanded by peptide were isolated by flow sorting, they (1×10^5^/100 µl) were inoculated into a 96-well plate immobilized with different dosage of peptide and incubated for 4 hours. CCK-8 reagent (10 µl) was added to each pore and continued to culture for 4 hours until the media turned yellow. The absorbance value (450 nm) of each pore was measured using a microplate reader. Data are shown as the mean of three independent experiments.

Since BP3 could specifically bind the BCG-specific γδ T cells, we infer it might contain a γδ TCR recognized epitope. A binding assay and amplification assay were performed, using PBMC isolated from human subjects inoculated with BCG or un-inoculated subjects, to investigate whether natural γδ T cells could recognize and become activated by the epitope peptide. Fresh PBMC were immobilized with anti-pan γδ TCR monoclonal antibody in 24-well culture plates with RPMI 1640 medium for 2 weeks to obtain enough numbers of γδ T cells. Flow cytometry was performed to analyze the binding capability of biotinylated BP3 with enriched γδ T cells from BCG vaccinated subjects and un-vaccinated subjects. Thirty-three percent of γδ T cells from human subjects inoculated with BCG bound to BP3; whereas only 5% γδ T cells from human subjects un-inoculated with BCG bound to BP3 (Figure 4A). In addition, immobilized BP3 were able to induce expansion of γδ T cells in PBMC from human subjects inoculated with BCG to an average 18% after co-culture for 2 weeks, significantly higher than the expansion to 6% γδ T cells in PBMC from human subjects un-inoculated with BCG (Figure 4B). The proliferation of γδ T cells expanded by BP3 peptide was further confirmed by the CCK-8 assay (Figure 4C). Taken together, these data demonstrate that the candidate epitope BP3 could bind and activate γδ T cells from human subjects inoculated with BCG *in vitro*.

### The immune functions of γ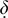 T cells from human subjects inoculated with BCG induced by peptide BP3

**Figure 5:**
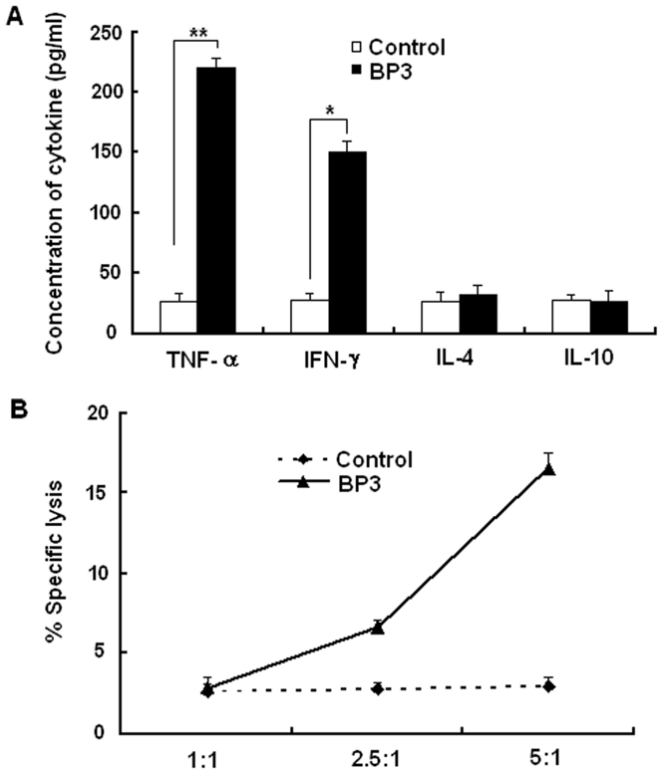
The immune function of γδ T cells from human subjects inoculated with BCG induced by peptide BP3. (A) The Th1 and Th2 cytokine production of γδ T cells induced by peptide BP3. After sorting the γδ T cells from human subjects inoculated induced by BP3, the Th1 (IFN-γ and TNF-α) and Th2 (IL-4 and IL-10) type cytokine production was detected by ELISA kit. Data are shown as the mean of pg/ml±S.D. of three independent experiments. The single asterisk denote significant difference (p<0.05). The double asterisks denote significant difference (p<0.01) (B) The cytotoxicity of γδ T cells induced by peptide BP3 to THP-1 infected with BCG. After sorting and culturing the γδ T cells from human subjects inoculated by BP3, MTT test was used to evaluate the cytotoxicity activities of γδ T cells to THP-1 cells infected with BCG in vitro. Data are shown as the mean of three independent experiments.

We determined the immune functions of γδ T cells from human subjects inoculated with BCG induced by peptide BP3 using cytokine production and cytotoxicity assays. A strong enhancing effect on Th1 cytokine (IFN-γ and TNF-α) production, but not Th2 cytokine (IL-4 and IL-10) production, was observed in γδ T cells from subjects inoculated with BCG and expanded by BP3 (Figure 5A). Then, we examined the cytotoxicity of γδ T cells induced by peptide to THP-1 cells infected by BCG. γδ T cells induced by peptide BP3 had a cytotoxic effect on THP-1 cells infected by BCG (Figure 5B).

### BLAST analysis

BLAST search was performed to identify motif-containing mycobacterial proteins in the sequences of 3 peptides. BLAST results are listed in Table 3. The peptide BP3 had the best matching characteristics to protein in *Mycobacterium*, with an E value of 39.2. The matching protein of BP3 was oxidative stress response regulatory protein (OXYS) in *M. tuberculosis K85*, *M. tuberculosis CPHL_A* and LysR family of transcriptional regulator in *M. tuberculosis KZN 4207*. Moreover, another BLAST analysis in all species demonstrated that there were no significant matches with other proteins. Meanwhile, further BLAST analyses confirmed that OXYS protein also exists in *M. bovis BCG str. Pasteur 1173P2*. It is conserved in BCG and many mycobacteria. Combining the above experimental results and bioinformatics analyses, the BP3 peptide was regarded as a promising candidate epitope peptide, which was recognized by the γδ TCR. In addition, OXYS protein was regarded as a candidate antigen recognized by the γδ TCR and selected for further analysis.

**Table 3:** Blast Analysis of BP1, BP2 and BP3.

No.	Reference	Protein	Species	E value	Matching part
BP1	ZP_06848846.1	stearoyl-CoA 9-desaturase	Mycobacterium parascrofulaceum	22	RH-S-W- PHD
BP1	ZP_05225536.1	Fatty acid desaturase	Mycobacterium intracellulare	22	RH-S-W- PHD
BP1	NP_959592.1	DesA1	Mycobacterium avium	22	RH-S-W- PHD
BP2	ZP_04748088.1	hypothetical protein MkanA1	Mycobacterium kansasii	34	LS- ELTLP
BP2	YP_890886.1	transcriptional regulator	Mycobacterium smegmatis	34	L- KEL- LPL
BP2	YP_952896.1	aldehyde oxidase and xanthine dehydrogenase	Mycobacterium vanbaalenii	34	L- KELT-LPL
*BP3*	ZP_06452924.1	oxidative stress response regulatory protein OXYS	Mycobacterium tuberculosis K85	6e-04	HPETQDSDDVD
*BP3*	ZP_06435391.1	oxidative stress response regulatory protein OXYS	Mycobacterium tuberculosis CPHL_A	6e-04	HPETQDSDDVD
*BP3*	ZP_05222720.1	LysR family transcriptional regulator	Mycobacterium tuberculosis KZN 4207	6e-04	HPETQDSDDVD

### The OXYS protein binds and activates γδ T cells *in vitro*

**Figure 6:**
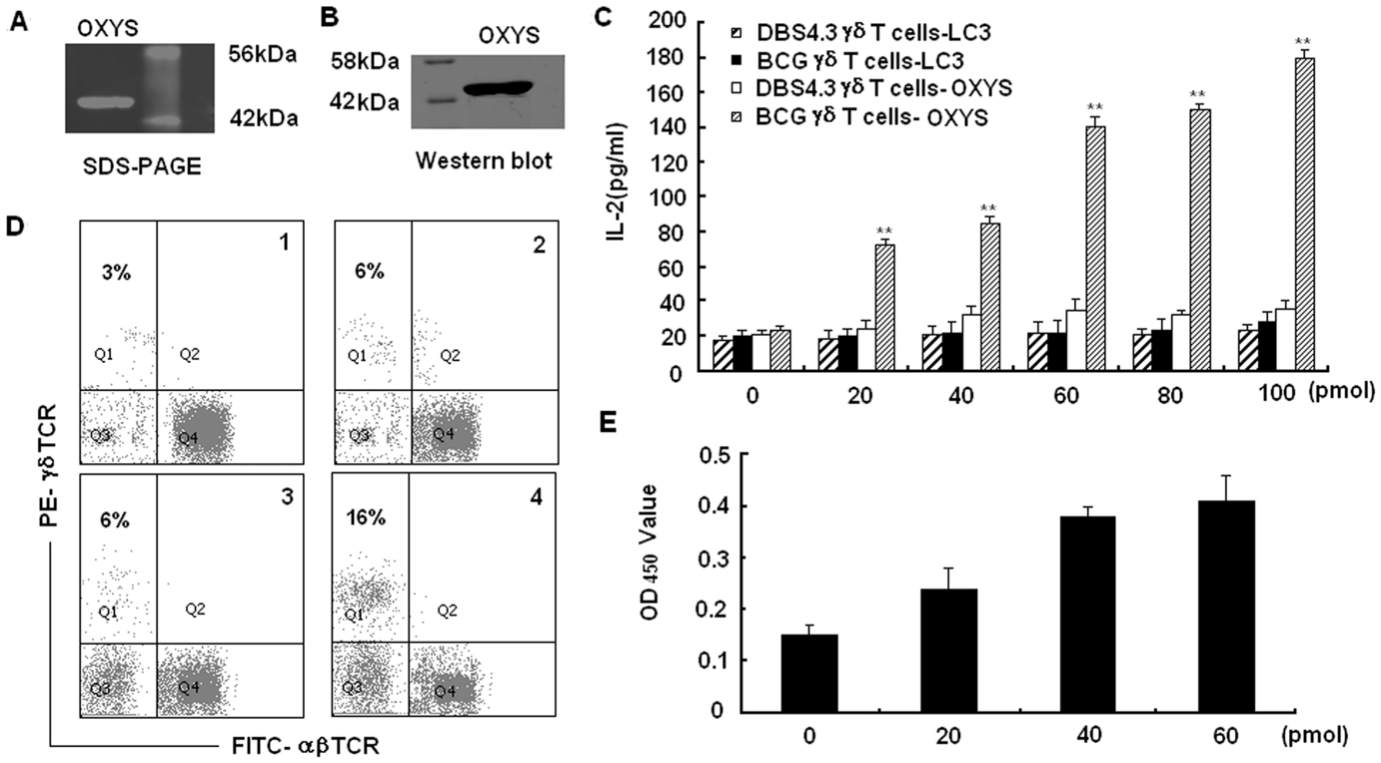
Binding and activating γδ T cells by OXYS protein *in vitro*. (A) The expression of OXYS protein in *E. coli* was confirmed by 12% SDS-PAGE. The molecular weight of the expressed OXYS is 44KD (B) The expression of OXYS protein was further confirmed by western blot using anti his-tag antibody. (C) OXYS protein could stimulate the BCG γδ T cells to produce IL-2 in a dose-dependent manner. LC3 protein, an autophagy detection marker was used as the control protein. Data are shown as the mean of pg/ml±S.D. of three independent experiments. The double asterisks represent significance difference (P<0.01). (D) OXYS protein could induce the expansion of γδ T cells from human subjects inoculated with BCG. Results are representative of three independent experiments. Panel 1 and panel 2 was the expansion percentage of γδ T cells from human subjects un-inoculated with BCG and inoculated with BCG induced by control protein, respectively. Panel 3 and panel 4 was the expansion percentage of γδ T cells from human subjects un-inoculated with BCG and inoculated with BCG induced by OXYS, respectively. (E) Detection of γδ T cell proliferation by CCK-8 assay. After the γδ T cells expanded by protein were isolated by flow sorting, they (1×10^5^/100 µl) were inoculated into a 96-well plate immobilized with different dosage of protein and incubated for 4 hours. CCK-8 reagent (10 µl) was added to each pore and continued to culture for 4 hours until the media turned yellow. The absorbance value (450 nm) of each pore was measured using a microplate reader. Data are shown as the mean of three independent experiments.

To date, there have been some controversies about whether γδ T cells could recognize intact protein, the recognition mechanism of γδ T cells to antigen remains uncertain. Although our study demonstrated BP3 could bind and activate γδ T cells, we had not yet determined whether its matched protein could be recognized by γδ T cells. To answer this question, we examined the binding activity of OXYS protein to transfected γδ T cells, as well as its ability to activate natural γδ T cells. We expressed the full-length OYXS protein with His tag in *E. coli*. The purification of OXYS protein was determined by SDS-PAGE (Figure 6A) and western blot with anti-His tag mAb (Figure 6B). The IL-2 secretion from BCG-specific γδ T cells and DBS4.3 γδ T cells after stimulation with purified OXYS protein at different concentrations was examined. Production of IL-2 in BCG-specific γδ T cells increased in a dose-dependent manner after simulation with OXYS protein (Figure 6C). Furthermore, exposure to OXYS protein induced expansion of γδ T cells in PBMC from human subjects inoculated with BCG to an average of 16% after co-culture for 2 weeks, significantly higher than the 6% γδ T cells in PBMC from human subjects un-inoculated with BCG (Figure 6D). The proliferation of γδ T cells expanded by OXYS protein was confirmed by the CCK-8 assay (Figure 6E). These data confirmed that OXYS protein expressed in *E. coli* could specifically bind to the γδ TCR, and activate γδ T cells from human subjects inoculated with BCG, suggesting γδ TCR might recognize intact protein as antigens.

## Discussion

In this study, we developed a novel strategy to identify epitopes and protein antigens recognized by γδ T cells by combining immunological and biochemical methods. With this strategy, we identified a promising γδ T cell-reactive BCG-specific epitope (peptide BP3) and one candidate BCG protein recognized by γδ T cells (protein OXYS). BCG-specific γδ TCR transfected cells could bind the peptide and could stimulate the expansion of γδ T cells from human subjects inoculated with BCG. Moreover, the BP3 matched mycobacterial protein OXYS was also bound by the BCG-specific γδ TCR transfected cells and *in vitro*, activated the γδ T cells from human subjects inoculated with BCG.

Previously, the γδ TCR transfected cells were used to evaluate the roles of some amino acids in antigen recognition [24]–[28]. This system could also be used to screen the antigens recognized by γδ T cells. Kong *et al.* [8] identified a γδ TCR recognized protein ULBP4 using the specific γδ TCR transfected cells. In our study, we first applied the transfected cells as probes to pan the phage library for screening of the epitopes recognized by γδ TCR. Prior to the panning process, we constructed the BCG-specific γδ TCR-transfected cells as tester cells and non-specific γδ TCR-transfected cells as driver cells. We performed 4 rounds of *in vitro* subtractive panning on tester and driver cells and developed some optimizing procedures to improve the probability of obtaining specific phages with high affinity. The binding specificity of BP3 and OXYS to the γδ TCR and their ability to activate transfected cell lines and natural γδ T cells proved the success of this method. The new method can be used to detect additional protein antigens recognized by γδ T cells in the future.

A potential problem of our research is the representativeness of the CDR3 sequences we used. The CDR3 sequences in BCG-expanded γ9/δ2 cells were obtained according to Spencer's report [11]. About 60% of the BCG-expanded γ9/δ2 cells contained the CDR3 sequence. Thus, the identified epitopes and proteins using cells transfected with this CDR3 sequence as a probe were specific to this subset of γ9/δ2 cells. However, due to the diversity of CDR3 sequences, the reported CDR3 sequence might not be the dominant sequence in different areas, people, or races. Therefore, the CDR3 sequence applied in our study might not be representative for all γ9/δ2 cells from human subjects inoculated with BCG, and the epitopes and proteins determined by this screening method might not fully represent antigens recognized by γδ T cells from human subjects inoculated with BCG. That might be the reason why peptide BP1 and BP2 could neither specifically bind to the transfected cell line expressing BCG-specific γδ TCR (Figure 3B), nor effectively activate γδ T cells isolated from human subjects inoculated with BCG (data not shown), even though they have a higher frequency of occurrence (Table 2).

As shown in figure 3A, all 3 peptides could stimulate more IL-2 production by DBS4.3 γδ T cells than control peptides. We believe it depends on the difference of DBS4.3 γδ TCR and BCG γδ TCR. Both TCRs differ only in the CDR3 sequence. They have common CDR1, CDR2, and fragment regions. Therefore, during the screening procedure, we did not guarantee that all peptides that partially bind driver cells and tester cells could be excluded. Although BP1, BP2, and BP3 could bind to BCG-specific γδ T cells and DBS4.3 γδ T cells, only the binding of BP3 to cells could be blocked by a functional γδ TCR blocking monoclonal antibody.

In this study, we identified one epitope peptide recognized by BCG-specific γδ TCR by screening the phD 12 phage-display peptide-library, but more peptide candidates probably remain unidentified. The phD 12 phage-display peptide-library was based on a combinatorial library of random peptide 12-mers fused to a minor coat protein of M13. The library contained a complexity of 2.7×10^9^ individual clones, representing the entire obtainable repertoire of 12-mer peptide sequences, which expresses a random 12 amino acid sequence. However, it still might not fully cover the sequence of BCG or *Mycobacterium*. More epitopes for the BCG-specific γδ TCR may be identified by screening the random expression genomic library of *Mycobacterium*.

The OXY protein identified as a γδ TCR recognized antigen according to this strategy, is a protein with a molecular mass of 44 kDa. This differs from the results of Boom, who found that protein antigens with a molecular mass of 10–14 kDa in the supernatant from *Mycobacterium tubercolosis* were a major stimulus for human γδ T cells [12]. However, the results of Boom did not exclude the possibility that other molecular mass of proteins may have an activation function for γδ T cells as well. According to our strategy, a distinct γδ T cell-recognized antigen was identified. Therefore, different screening methods may be complementary to each other to explore new protein antigens recognized by the γδ TCR.

We compared γδ T cells expanded by BP3 peptide or OXYS protein obtained from un-vaccinated persons and BCG vaccinated persons. The results showed that immobilized BP3 was able to induce the expansion of γδ T cells in PBMC from human subjects inoculated with BCG to an average 18%, significantly higher than the 6% expansion of γδ T cells in PBMC from human subjects un-inoculated with BCG. Meanwhile, OXYS protein was able to induce expansion of γδ T cells in PBMC from human subjects inoculated with BCG to an average 16%, significantly higher than the 6% expansion of γδ T cells in PBMC from human subjects un-inoculated with BCG. In addition to assessing the proliferation of γδ T cells by percentages, we also used the CCK-8 assay to detect the proliferation of γδ T cells. Cell Counting Kit-8 allows sensitive colorimetric assays for the determination of the number of viable cells in cell proliferation and cytotoxicity assays. CCK-8 was reduced by dehydrogenases in cells to give a yellow colored product (formazan), which is soluble in the tissue culture medium. The amount of the formazan dye generated by the activity of dehydrogenases in cells is directly proportional to the number of living cells. So according to the change of the absorbance, the data did show the proliferation of BP3 and OXYS stimulated γδ T cells.

In conclusion, our results provide new insights about γδ T cells and BCG antigen from 3 aspects. First, we provided a novel strategy to identify epitopes and proteins recognized by γδ TCR. The new strategy could be expanded to the use of detecting other protein antigens recognized by γδ T cells. Second, our findings added to the understanding of the recognition mechanism of γδ TCR to protein antigens. Third, the peptide BP3 or OXYS protein were identified as potential candidate peptides or antigenic components to develop new tuberculosis vaccines or adjuvant, as well as biomarkers of tuberculosis infection. Further studies are in progress to confirm the bio-function of the target peptide and OXYS protein in anti-tuberculosis infection.

## Materials and Methods

### Ethics Statement

This work received approval from the Clinical Ethics Committee of the Institute of Pathogen Biology, Chinese Academy of Medical Sciences and Peking Union Medical College. All subjects gave their informed consent to participate. The consent was verbal. The ethics committee specifically approved that procedure.

### Subjects

Twenty volunteers composed of 12 men and 8 women aged 15 to 32 years were enrolled in this study. Of these, 15 subjects were vaccinated with BCG and 5 subjects were not vaccinated with BCG. Exclusion criteria for all subjects were smoking, medication, pregnancy and any abnormalities in renal and liver function tests.

### Reagents and cell lines

The phD 12 phage-display peptide-library kit (New England Biolabs, USA) was used to screen specific peptides binding to γδ TCR. The titer of the library is 2×10^13^ pfu. The *E. coli* host strain ER2738 was used for M13 phage propagation. J.RT3-T3.5 cells and THP-1 cells, a human myelomonocytic cell line, were obtained from the American Type Culture Collection (ATCC). These cells and the transfected cells derived from them were maintained in RPMI-1640 supplemented with 10% FCS, penicillin, streptomycin, and 5×10^−5^ M β-mercaptoethanol. γδ T cells were obtained from fresh peripheral blood mononuclear cells (PBMC) by immobilization by anti-pan-γδ TCR monoclonal antibody (Immunotech). In brief, PBMC was separated from peripheral blood by density gradient centrifugation on Ficoll-Hypaque (GE Healthcare). The cells were grown in RPMI 1640 medium supplemented with 12% FCS, 200 U/mL IL-2, penicillin, streptomycin, and 5×10^−5^ M β-mercaptoethanol, in a 24-well cell culture plate containing immobilized anti-pan-γδ TCR monoclonal antibody at 37°C in 5% CO_2_. Every 3–4 days, half of the medium was discarded and replenished by fresh medium and cytokines. After 2 weeks of culture, the viability of cells assessed by flow cytometry using 7AAD stain was more than 90%. The purified cell population contained about 80% viable γδ T cells.

### Construction of transfected cells expressing BCG-specific and non-specific γ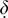 TCRs

A full-length γ9 or δ2 chain was amplified from PBMC cDNA using specific primers containing *Kpn*I and *Xho*I restriction sites. The BCG-specific CDR3 sequence was inserted into full-length γ9 and δ2 chain to substitute for the original CDR3 sequence using overlapping PCR. The full-length TCR chain with BCG-specific CDR3 sequence was digested with *Kpn*I and *Xho*I and cloned into pREP7 and pREP9 expression vectors with either hygromycin or neomycin resistance. Meanwhile, full-length pREP7-γ9 and pREP9-δ2 chains of DBS4.3, a known γδ T-cell clone, with a different γ9 and δ2 CDR3 sequence than the BCG-specific γδ TCR, was also constructed according to same procedure, as a non-BCG specific control. The J.RT3-T3.5 cells (1.2×10^7^) were co-transfected with 20 µg of pREP7-γ9 and pREP9-δ2 by electroporation at 260 V and 975 µF using Bio-Rad Gene-Pulser. After 48 h, the transfected cells were cultured in selection medium with hygromycin and neomycin for 4 weeks. The resulting cells expressing surface γδ TCR were evaluated by flow cytometry with fluorescein isothiocyanate (FITC)-conjugated anti-human γ9 Biolegend and phycoerythrin (PE)-conjugated anti-human δ2 (Biolegend) monoclonal antibody. The double positive cells were isolated by flow sorting for further experiments (BD FACSAria I).

### *In vitro* panning

The transfected cells expressing BCG-specific γδ TCR (named BCG γδ T cells) were used as tester cells, and the transfected cells expressing DBS4.3 γδ TCR (named DBS4.3 γδ T cells) were used as the driver cells for subtractive screening from a phage-display 12-peptide library. The driver cells were washed with phosphate buffered saline (PBS) and kept in serum-free RPMI-1640 for 1 h before blocking with 3 mL blocking buffer (5% bovine serum albumin (BSA) in PBS) for 30 min at 37°C. Approximately 2×10^11^ pfu phages were added to the blocked driver cells and mixed gently for 1 h at 37°C. The cells were then pelleted by centrifugation at 800 rpm for 5 min. The supernatants containing the phages were incubated with the blocked tester cells for 1 h at 37°C before the cells were pelleted again. The cells were washed twice with 0.1% TBST (50 mM Tris-HCl, pH 7.5, 150 mM NaCl, 0.1% Tween-20) to remove unbound phage particles. The tester cells and bound phages were both incubated with *E. coli* host strain ER2738 and the phages were rescued by infection with bacteria when the cells died. The phage titer was subsequently evaluated by a blue plaque-forming assay on agar plates containing tetracycline. Finally, a portion of purified phage preparation was used as the input phage for the next round of *in vitro* selection. In total, 4 rounds of selection were taken. For each round of selection, 2×10^11^ pfu of collected phages were used. The panning intensity was increased by prolonging the phages incubation process with driver cells to 1.25 h, 1.5 h, and 2 h and by shortening the process with tester cells to 45 min, 30 min, and 15 min in the second, third, and fourth rounds of selection, respectively. Meanwhile the number of TBST washes was increased to 4, 6, and 8 times in the second, third, and fourth rounds of selection, respectively.

### Identification for phage clones binding to transfected cells by phage-ELISA, PCR and sequencing

The tester cells and driver cells were seeded into 96-well plates (1×10^5^ cells/well) one day before use and then fixed in 96-well plates, with 4% formaldehyde for 15 min at room temperature. The plates were washed 3 times with PBS. The amplified phage clones were then picked out randomly and blocked individually with 1% BSA at 37°C for 30 min. These phage clones were added into the tester and driver cells (1×10^10^ pfu/well) and incubated at 37°C for 2 h. The plates were washed 3 times with PBS, and then the mouse anti-M13 phage antibody was added at a dilution of 1∶1000 in TBST and incubated at 37°C for 1 h. After reaction with HRP-conjugated goat anti-mouse IgG antibody (eBioscience) and substrate (Sigma), the plates were read on a microplate reader (SpectraMax M5) at 450 nm. As negative controls, PBS and unrelated phages with the same titer were used. The positive phage clones in ELISA were amplified by PCR, and the parts containing epitopes were sequenced using the 3730 sequencer with the sequencing primer, 5′-CC CTC ATA GTT AGC GTA ACG-3′ (supplied in the kit).

### Peptide synthesis and labeling

After *in vitro* screening and sequencing confirmation, 3 candidate peptides BP1, BP2 and BP3 were selected for further analysis and were synthesized in the peptide synthesis facility of the Academy of Military Medical Sciences, China. The purity of synthesized peptides was more than 90% in HPLC analysis. Half of the synthesized peptides were labeled with biotin at their N terminal ends.

### Protein expression and preparation of BCG soluble extracts

Purified oxidative stress response regulatory protein (oxyS, Rv0117) PCR fragments amplified from H37Rv genomic were digested with *Kpn*I and *Xho*I enzymes and linked into prokaryotic expression plasmid pET30a. Freshly transformed *E. coli* BL21 (DE3) cells harboring plasmid pET30a-oxyS were cultured in 500 mL of LB medium containing kanamycin at 37°C. When the cell density reached 0.8–1.0 (OD600), isopropyl-β-D-thiogalactopyranoside (IPTG, Sigma) was added to a final concentration of 1 mM, and the bacteria were cultured for another 3 h at 37°C. The culture was then harvested and centrifuged at 5000×*g* for 15 min at 4°C. The inclusion body was dissolved in 8 M urea and then purified with Amersham Biosciences HisTrap column. The His-tagged protein molecular weight and purity were confirmed by SDS-PAGE on 12% acrylamide gels and western blot. The BCG bacteria were heat inactivated at 85°C for 20 min, and then the bacteria were subjected to 5 h of sonification at room temperature in a water bath sonicator. The extracts were then spun at 13000×*g* for 5 min and the supernatants were collected.

### Binding of J.RT3-T3.5 transfectants to peptides and proteins

The transfected cells were pre-incubated with 10 ng/mL Phorbol Myristate Acetate (PMA) at room temperature for 30 min. After extensive washes with RPMI-1640, the cells were plated into 24-well plates at 10^6^ per well in the presence of 3 peptides, control peptide, OXYS protein, control protein or BCG soluble extracts. The supernatants were harvested after 24 h and the level of IL-2 was detected using the Human IL-2 ELISA Kit (B.D.Company) according to the manufacturer's instructions. The binding of transfected cells to peptides was also detected via flow cytometry. The cells were incubated with biotin-conjugated peptide or control peptide and FITC-conjugated streptavidin (eBioscience) was added, and the cells were incubated for 30 min at 4°C. The cells were then analyzed by flow cytometry.

### Binding of γδ T cells from BCG vaccinated and un-vaccinated persons to peptides

Fresh PBMC from BCG vaccinated and un-vaccinated persons in 24-well culture plates with RPMI 1640 medium were immobilized with anti-pan γδ TCR monoclonal antibody for 2 weeks to obtain enough amounts of γδ T cells. The cells incubated with biotin-conjugated peptide or control peptide and FITC-conjugated streptavidin. The cells were analyzed by flow cytometry.

### Peptide/protein-immobilized amplification assay

PBMC from subjects vaccinated with BCG and un-vaccinated subjects were immobilized with peptides or OXYS protein for the amplification assay. The PBMC were cultured in RPMI 1640 medium with 10% FCS and IL-2 (200 U/mL) for 2 weeks and then the percentage of γδ T cells was measured by FACS analysis. The positive γδ T cells were isolated by flow sorting for further functional experiments.

### Determination of γδ T cells proliferation

The proliferation of γδ T cells was detected by the Cell Counting Kit-8 (CCK-8) assay (KeyGEN). After the γδ T cells expanded by peptide and protein immobilization were isolated by flow sorting, 1×10^5^/100 µL were inoculated into a 96-well plate, immobilized with different dosages of peptide/protein, and incubated for 4 h. CCK-8 reagent (10 µL) was added to each well and culture was continued for 4 h until the media turned yellow. The absorbance value at 450 nm of each well was measured using a microplate reader (SpectraMax M5).

### Cytokine production

After the γδ T cells activated by BP3 peptide were sorted, the production of cytokines IL-4, IL-10, TNF-α and IFN-γ were determined by ELISA according to the manufacturer's instructions.

### MTT cytotoxicity

The 3-(4,5-dimethylthiazol-2-yl)-2,5-diphenyltetrazolium bromide (MTT) colorimetric test was used to evaluate the cytotoxic effects of γδ T cells on THP-1 cells infected with BCG *in vitro*. The BCG strain was grown in Middlebrook 7H9 liquid medium (Invitrogen) with 10% albumin/dextrose-catalase (ADC) enrichment media (sigma) supplemented with 0.2% glycerol. THP-1 cells were infected with BCG strains at a multiplicity of infection (MOI) of approximately 10 bacterial cells per macrophage. THP-1 cells infected by BCG were used as target cells and seeded into 96-well plates at 10^4^ cells per well. The γδ T effector cells were added to each well with an appropriate ratio of effector cells and target cells. After the effector cells and the target cells were incubated at 37°C for 4 h, 15 µL of MTT solution (5 mg/mL) was added to each well, and incubated at 37°C for an additional 4 h. The reaction was stopped by the addition of 100 µL dimethyl sulfoxide to dissolve the tetrazolium crystals. The plate was examined at 570/630 nm on a microplate reader (SpectraMax M5) and the percentage of specific lysis was calculated.

### Bioinformatics analysis

Homologous analysis and sequence alignment were done using BLAST (Basic Local Alignment Search Tool) programs to determine matched protein of related peptides. In the NCBI website http://www.ncbi.nlm.nih.gov/blast, the program of protein BLAST was selected for bioinformatics analysis. The amino acid sequences of BP1, BP2, and BP3 were input, the non-redundant protein sequence (nr) was chosen in the database and the organism was limited to *Mycobacterium*. Three matched proteins with higher scores were listed. To demonstrate that the matched protein was specific for *Mycobacterium*, we performed another blast analysis in which the organism was limited in all species.

### Statistical analysis

Statistical comparisons were performed using the Student's *t* test. A value of P<0.05 was considered statistically significant. In BLAST analysis, the corresponding E-value for each score was computed. The smaller the E-value, the greater the belief that the aligned sequences are homologous.

## Supporting Information

Table S1 The sequences of specific primers used to produce full length BCG-specific and DBS4.3 γ9/δ2 chains are listed in this file.
(DOC)

## References

[1] Hiromatsu K, Yoshikai Y, Matsuzaki G, Ohga S, Muramori K (1992). A protective role of γ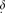 T cells in primary infection with listeria monocytogenes in mice.. J Exp Med.

[2] Balbi B, Valle MT, Oddera S, Giunti D, Manca F (1993). T-lymphocytes with γδ Vδ2 antigen receptors are present in increased proportions in a fraction of patients with tuberculosis or with sarcoidosis.. Am Rev Respir Dis.

[3] Martino A, Casetti R, Sacchi A, Poccia F (2007). Central Memory Vγ9Vδ2 T Lymphocytes Primed and Expanded by Bacillus Calmette-Gue'rin-Infected Dendritic Cells Kill Mycobacterial-Infected Monocytes.. J Immunol.

[4] Lee J, Choi K, Olin MR, Cho SN, Molitor TW (2004). γδ T Cells in Immunity Induced by Mycobacterium bovis Bacillus Calmette-Gue'rin Vaccination.. Infect Immun.

[5] Morita CT, Lee HK, Leslie DS, Tanaka Y, Bukowski JF (1999). Recognition of nonpeptide prenyl pyrophosphate antigens by human γδ T cells.. Microbes and Infection.

[6] Nishimura H, Hirokawa M, Fujishima N, Fujishima M, Miura I (2004). Contribution of Complementarity-Determining Region 3 of the T Cell Receptor Vδ2 Chain to the Recognition of Aminobisphosphonates by Human γδ T-Cell.. Int J Hematol.

[7] Cao W, He W (2005). The recognition pattern of γδ T cells.. Front Biosci.

[8] Kong Y, Cao W, Xi XY, Ma C, Cui LX (2009). The NKG2D ligand ULBP4 binds to TCR γ9/δ2 and induces cytotoxicity to tumor cells through both TCR γδ and NKG2D.. Blood.

[9] Chen H, He X, Wang Z, Wu D, Zhang H (2008). Identification for human TCRγδ-recognized epitopes/proteins via CDR3δ peptide-based immuno-biochemical strategy.. J Biol Chem.

[10] Scotet E, Martinez LO, Grant E, Barbaras R, Jenö P (2005). Tumor Recognition following Vγ9Vδ2 T Cell Receptor Interactions with a Surface F1-ATPase-Related Structure and Apolipoprotein A-I.. Immunity.

[11] Spencer CT, Abate G, Blazevic A, Hoft DF (2008). Only a subset of phosphoantigen-responsive γδ T cell mediated protective tuberculosis immunity.. J Immunol.

[12] Boom WH, Balaji KN, Nayak R, Tsukaguchi K, Chervenak KA (1994). Characterization of a 10- to 14-kilodalton protease-sensitive Mycobacterium tuberculosis H37Ra antigen that stimulates human γδ T cells.. Infect Immun.

[13] Wang J, Li BQ (2009). Phenotype expression and function of antigen presenting cells in human γδ T cells activated by peptide antigen from Mycobacterium tuberculosis.. Xi Bao Yu Fen Zi Mian Yi Xue Za Zhi.

[14] Zhang Ruijun, Zheng Xiaodong, Li Baiqing, Wei Haiming, Tian Zhigang (2006). Human NK Cells Positively Regulate γδ T Cells in Response to Mycobacterium tuberculosis.. J Immunol.

[15] Price SallyJ, Hope JayneC (2008). Enhanced secretion of interferon-γ by bovine γδ T cells induced by coculture with Mycobacterium bovis-infected dendritic cells: evidence for reciprocal activating signals.. Immunology.

[16] Brandes M, Willimann K, Bioley G, Levy N, Eberl M (2009). Cross-presenting human γδ T cells induce robust CD8+ αβ T cell responses.. Proc Natl Acad Sci U S A.

[17] Li Li, Wu Chang-You (2008). CD4+CD25+Treg cells inhibit human memory γδ T cells to produce IFN-γ in response to M tuberculosis antigen ESAT-6.. Blood.

[18] Barry MA, Dower WJ, Johnston SA (1996). Toward cell-targeting gene therapy vectors: selection of cell-binding peptides from random peptide-presenting phage libraries,. Nature Med.

[19] Brown KC (2000). New approaches for cell-specific targeting: identification of cell-selective peptides from combinatoriallibraries,. Curr Opin Chem Biol.

[20] Zhang BH, Zhang YQ, Wang JW, Zhang YD, Chen JJ (2007). Screening and Identification of a Targeting Peptide to Hepatocarcinoma from a Phage Display Peptide Library.. Mol Med.

[21] Alderson MR, Bement T, Day CH, Zhu LQ, Molesh D (2000). Expression cloning of an immunodominat family of Mycobacterium tuberculosis antigen using human CD4+T cells.. J Exp Med.

[22] Xu CP, Zhang HY, Hu HB, He HB, Wang Z (2007). γδ T cells recognize tumor cells via CDR3 region.. Mol Immunol.

[23] Xi XY, Guo Y, Chen H, Xu CP, Zhang HY (2009). Antigen specificity of γδ T cells primarily depends on the flanking sequences of CDR3δ.. J Bio Chem.

[24] Xi XY, Cui LX, He W (2010). The recognition of γδ TCR to protein antigen does not depend on the hydrophobic I97 residue of CDR3δ.. Int Immunol.

[25] Bukowski JF, Morita CT, Tanaka Y, Bloom BR, Brenner MB (1995). Vγ2Vδ2 TCR-Dependent Recognition of Non-Peptide Antigens and Daudi Cells Analyzed by TCR Gene Transfer.. J Immunol.

[26] Miyagawa F, Tanaka Y, Yamashita S, Mikami B, Danno K (2001). Essential Contribution of Germline-Encoded Lysine Residues in Jr1.2 Segments to the Recognition of Nonpeptide Antigens by Human γδ T cell.. J Immunol.

[27] Morita CT, Lee HK, Wang H, Li H, Mariuzza RA (2001). Structural Features of Nonpeptide Prenyl Pyrophosphates That Determine Their Antigenicity for Human γδ T Cells.. J Immunol.

[28] Morita CT, Beckman EM, Bukowski JF, Tanaka Y, Band H (1995). Direct presentation of nonpeptide prenyl pyrophosphate antigens to human γδ T cells.. Immunity.

