# Supplementary material for: A Novel Strategy to Screen Bacillus Calmette-Guérin Protein Antigen Recognized by γδ TCR
Source: PLoS One. 2011 Apr 22;6(4):e18809. doi: 10.1371/journal.pone.0018809 (PMC3081299; doi:10.1371/journal.pone.0018809)
Supplement: Table S1 — The sequences of specific primers used to produce full length BCG-specific and DBS4.3 γ9/δ2 chains are listed in this file. (DOC) [file pone.0018809.s001.doc]

Table 1 Primer sequence used to construct DBS4.3 and BCG specific full-length γ9 and δ2 chains

| Name | Primer Sequence |
| --- | --- |
| 1.DBSγ9−up | 5’-CGGGGTACCATGCTGTCACTGCTCCACAC-3’ |
| 2.DBS γ9−FP | 5’-GGAGTGGGAGTTGGGCAAAAAAATCAAGGTATTTGGTCCCGGAA-3’ |
| 3.DBSγ9−RP | 5’-TTTTTTTGCCCAACTCCCACTCCCACAAGGCACAGTAGTA-3’ |
| 4.DBSγ9−down | 5’-CTCGAGTCATCATGATTTCTCTCCAT-3’ |
| 5.DBSδ2−up | 5’-CGGGGTACCATGCAGAGGATCTCCTCCCTC-3’ |
| 6.DBSδ2−FP | 5’-ATCGGTACTCACTAGCGTGTCACAGGCACAGTAGTAAGACCCTTC-3’ |
| 7.DBSδ2−RP | 5’-TGTGACACGCTAGTGAGTACCGATAAACTCATCTTTGGAAAAGGA-3’ |
| 8.DBSδ2−down | 5’-CCGCTCGAGTTACAAGAAAAATAACTTGGCAGTC-3’ |
| 9.BCGγ9-FP | 5’-TACTGTGCCTTGTGGGAGGTGATTTCTGAGTTGGGC-3’ |
| 10.BCGγ9-RP | 5’-CTTGATTTTT TTGCCCAACT CAGAAATCAC CTCCCACAA-3’’ |
| 11.BCGδ2-FP | 5’-GGTTCCTACGTTCCTACCGGGGAGACCGATAAACTCATCTTTGG-3’ |
| 12.BCGδ2-RP | 5’-CGGTAGGAACGTAGGAACCCACGGTGTCACAGGCACAGTAGTAAG-3’ |

up: Upstream primer

down: Downstream primer

FP: Forward primer

RP: Reverse primer
